# Analysis of Lipid Peroxidation by UPLC-MS/MS and Retinoprotective Effects of the Natural Polyphenol Pterostilbene

**DOI:** 10.3390/antiox10020168

**Published:** 2021-01-23

**Authors:** Isabel Torres-Cuevas, Iván Millán, Miguel Asensi, Máximo Vento, Camille Oger, Jean-Marie Galano, Thierry Durand, Ángel L. Ortega

**Affiliations:** 1 Neonatal Research Group, Health Research Institute La Fe, Av. Fernando Abril Martorell 106, 46026 Valencia, Spain; maria.i.torres@uv.es (I.T.-C.); imiya@alumni.uv.es (I.M.); maximo.vento@uv.es (M.V.); 2 Department of Physiology, Faculty of Pharmacy, University of Valencia, Vicente Andrés Estellés Av. s/n, 46100 Burjassot, Spain; Miguel.A.Asensi@uv.es; 3 Institut des Biomolécules Max Mousseron, IBMM, University of Montpellier, CNRS, ENSCM, 34093 Montpellier, France; camille.oger@umontpellier.fr (C.O.); jean-marie.galano@umontpellier.fr (J.-M.G.); thierry.durand@umontpellier.fr (T.D.)

**Keywords:** diabetic retinopathy, lipid peroxidation, oxidative stress, polyphenols, pterostilbene, biomarker

## Abstract

The loss of redox homeostasis induced by hyperglycemia is an early sign and key factor in the development of diabetic retinopathy. Due to the high level of long-chain polyunsaturated fatty acids, diabetic retina is highly susceptible to lipid peroxidation, source of pathophysiological alterations in diabetic retinopathy. Previous studies have shown that pterostilbene, a natural antioxidant polyphenol, is an effective therapy against diabetic retinopathy development, although its protective effects on lipid peroxidation are not well known. Plasma, urine and retinas from diabetic rabbits, control and diabetic rabbits treated daily with pterostilbene were analyzed. Lipid peroxidation was evaluated through the determination of derivatives from arachidonic, adrenic and docosahexaenoic acids by ultra-performance liquid chromatography coupled with tandem mass spectrometry. Diabetes increased lipid peroxidation in retina, plasma and urine samples and pterostilbene treatment restored control values, showing its ability to prevent early and main alterations in the development of diabetic retinopathy. Through our study, we are able to propose the use of a derivative of adrenic acid, 17(*RS*)-10-*epi*-*SC*-Δ^15^-11-dihomo-IsoF, for the first time, as a suitable biomarker of diabetic retinopathy in plasmas or urine.

## 1. Introduction

Diabetes is a disease with high social impact and multiple systemic consequences. The prevalence of this disease has increased from 285 million to 463 million in the last decade and is expected to rise to 700 million in 2045 [1,2]. However, many diabetic cases are not strictly controlled until the pathology is at an advanced stage, with the loss of metabolic control leading to the development of long-term damages related to neurological, micro- and macrovascular alterations in different body organs, such as the retina. In fact, diabetic retinopathy (DR) is the most frequent ocular pathology caused by diabetes and the main cause of preventable blindness cases in economically active people (20–65 years) [3].

The presence of the blood-retina barrier means the retina is well protected from the leakage of circulating blood toxins. However, this tissue is extremely sensitive to alterations in oxygen levels [4]. Under chronic hyperglycemia, redox homeostasis is severely impaired and the overproduction of reactive oxygen species triggers neuronal and vascular damage, critical processes in the DR development [3]. Hyperglycemia induces alterations in biochemical pathways such as the polyol pathway, increased flux of advanced glycation end products/receptors (AGE/RAGE), and the hexosamine pathway which contributes to an oxidizing environment and triggers a state of low chronic inflammation leading to progression of DR to advanced stages [3]. Moreover, the delay in the treatment of diabetes together with the accumulated molecular damage inflicted by oxidative stress enables the phenomenon of metabolic memory, which refers to the development of DR even though there is good late glycemic control [5].

Polyphenols are secondary metabolites of plants with high antioxidant properties. In fact, the benefit of polyphenols has been suggested for combatting diabetes complications such as DR [3,6,7]. Pterostilbene (Pter), a natural analog of resveratrol, has recently been proposed as an inhibitor of aldose reductase, the rate-limiting enzyme of the polyol pathway, and AGE production [8], besides, it was able to reduce the levels of hexose and hexosamine in plasma, hepatic, and renal tissues to control values in diabetic animals [9], showing the polyphenol as a possible therapeutic agent against long-term complications of diabetes. Our group showed that parenteral daily administration of Pter, trans-3,5-dimethoxy-4′-hydroxystilbene, protected alloxan-induced diabetic rabbits by attenuating hyperglycemia-mediated oxidative stress via PI3K/AKT/GSK3β/NRF2 pathway [7]. The stilbene reduced lipid and protein oxidative damage, stimulated the activity of superoxide dismutase, catalase and glutathione peroxidase and increased GSH levels in the retina with a reduction in oxidative stress preventing early DR alterations [7].

The retina is one of the body tissues with the highest concentration of long-chain polyunsaturated fatty acids (PUFAs), the latter being essential for the maintenance of the physiological retinal function and development [10,11]. There is increasing evidence of the importance of lipids as mediators in the development of different retinal pathologies. In fact, the alteration of the lipid profile of patients is determinant in the progression of DR [12] and lipid peroxidation end product such as 4-hydroxy-2,3-trans-nonenal (4-HNE) alters the lysosomal functionality generating lipofuscin accumulation [13]. Based on these evidences, we hypothesize that Pter reverses the lipid oxidation profile induced in retinas under chronic hyperglycemia in vivo and that these changes may be reflected in plasma and urine becoming a potential biomarker for early detection of pathology.

## 2. Materials and Methods

### 2.1. Animal Model

In this study, male New Zealand rabbits obtained from Granja San Bernardo, Navarra, Spain were used. All procedures were performed according to established criteria by the Ethics Committee for Animal Experimentation and Welfare of the University of Valencia (Spain) (ethical code number of animal use 2016/VSC/PEA/00198, Generalitat Valenciana). Housing conditions and experimental procedures were in accordance with European Union (Directive 2010/63/EU) and Spanish (Royal Decree 53/2013) regulations.

Rabbits were randomly divided into 3 groups: control (non-diabetic), diabetic (diabetic), and treated diabetic (treated). In the experimental groups (diabetic and treated), diabetes was induced with alloxan (Sigma-Aldrich, St. Louis, MO, USA) following the protocol described by Alabadí et al. [14]. Briefly, animals were sedated with intramuscular injection of ketamine (Ketalar^®^, Pfizer Inc., Richmond, VA, USA) (35 mg/kg) and xylazine (Dechra Pharmaceuticals PLC, Northwich, UK) (5 mg/kg). Next, diabetes was induced by injecting alloxan (100 mg/kg) into the marginal ear vein. In addition, to prevent hypoglycemia, 5% glucose (Sigma-Aldrich, St. Louis, MO, USA) (10 mL) was administered intravenously, and drinking water was supplemented with 10% glucose for 24 h. In the case of the group of treated rabbits, Pter treatment began 48 h after inducing diabetes. The isotonic solution of Pter phosphate disodium salt (Syncom, Groningen, The Netherlands) was subcutaneously administered to treated diabetic animals daily (74 mg/kg which is equal to 50 mg/kg of Pter). Animals were euthanized by intravenous administration of sodium pentobarbital (Vetoquinol, Madrid, Spain) (100 mg/kg) 6 weeks after diabetes induction.

### 2.2. Standards and Reagents

Methanol, NaOH, KOH, ethanol, heptane, acetonitrile and ethyl acetate (analytical grade) were purchased from Sigma (Sigma-Aldrich, St. Louis, MO, USA). The formic acid and HCl were from Panreac (Barcelona, Spain) and Ultrapure H_2_O was generated on a milliQ system from Millipore.

Lipid oxidative damage was determined by the analysis of prostaglandines (PG), isoprostanes (IsoP), neuroprostanes (NeuroP), dihomo-IsoP, and dihomo-isofurans (dihomo-IsoF) using ultra high-performance liquid chromatography mass spectrometry (UPLC-MS/MS) (Waters Acquity UPLC-XevoTQ system, Milford, MA, USA). The analysis was carried out on different biological matrices: retina, plasma and urine.

**Figure 1:**
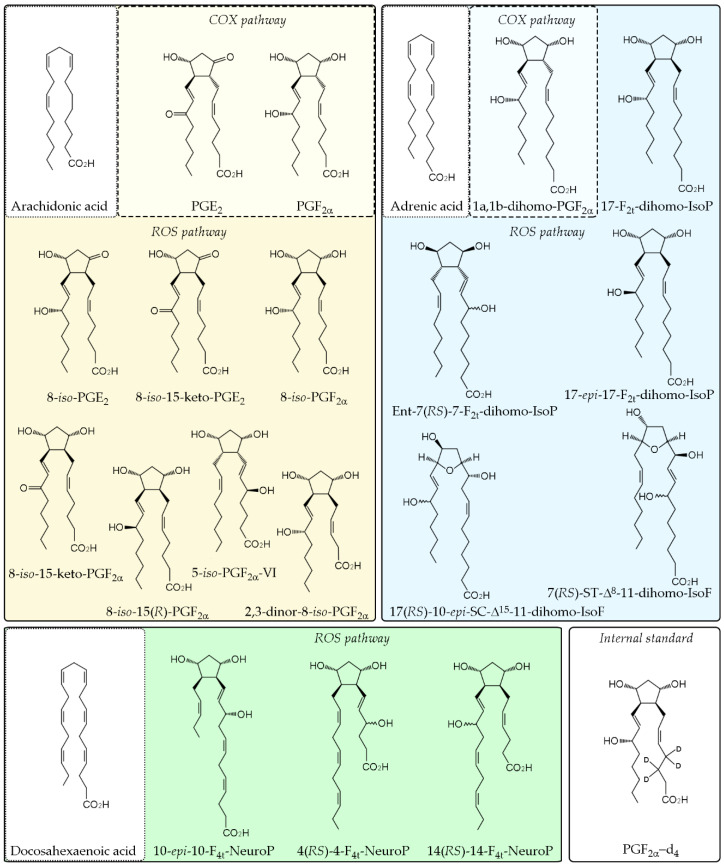
Chemical structure of analytes and internal standard. The analyzed PUFAs derived from arachidonic acid, adrenic acid and docosahexaenoic acid through cyclooxygenase (COX) pathway and/or non-enzymatic peroxidation are separated in different color boxes.

The prostaglandin standard PGE_2_, PGF_2α_, the IsoP standards 8-*iso*-PGE_2_, 8-*iso*-15-keto-PGE_2_, 8-*iso*-PGF_2α_ (also named 15-F_2t_-IsoP), 8-*iso*-15-keto-PGF_2α_, 8-*iso*-15(R)-PGF_2α_, 5-iso-PGF_2α_–VI, 2,3-dinor-8-iso-PGF_2α_, 1a,1b-dihomo-PGF_2α_, and deuterated internal standard PGF_2α_-d_4_ were obtained from Cayman Chemical Company (Ann Arbor, MI, USA) (Figure 1). The standards 10-*epi*-10-F_4t_-NeuroP, 4(*RS*)-4-F_4t_-NeuroP, 14(*RS*)-14-F_4t_-NeuroP, 17-F_2t_-dihomo-IsoP, Ent-7(*RS*)-7-F_2t_-dihomo-IsoP, 17-*epi*-17-F_2t_-dihomo-IsoP, 17(*RS*)-10-*epi*-*SC*-Δ^15^-11-dihomo-IsoF, 7(*RS*)-ST-Δ^8^-11-dihomo-IsoF were synthesized at the Institute of Biomolecules Max Mousseron (IBMM) (Montpellier, France) by Professor Durand’s team [15] (Figure 1).

### 2.3. Retina Sample Analysis

Animals were sacrificed as mentioned above, by intravenous administration of sodium pentobarbital. Subsequently, ocular enucleation was performed, and the retinas were separated into two halves and stored in cryotubes at −80 °C until further analysis. Approximately 30–50 mg of retina was stored for 30 min at 42 °C with 1 mL of methanolic NaOH (12 g of NaOH, 23 mL of H_2_O and 160 mL of MeOH). Then, they were homogenized and transferred to a glass tube, in which 400 µL of homogenate, 800 µL of H_2_O, 400 µL of methanolic NaOH and 5 µL of internal standard (PGF_2α_-d_4_ 20 µM) were added, and then heated for 45 min at 42 °C to produce saponification. The samples were then cooled on ice for 10 min. Subsequently, for protein precipitation, the pH of the sample was adjusted to 3 with HCl (3 M) and centrifuged at 3000× *g*. Once the supernatant was obtained, L/L extractions were carried out to extract the IsoP with 3 mL of hexane. It was centrifuged again for 5 min at 3000× *g*. The organic phase was removed, and 3 mL of ethyl acetate were added to the IsoP extract and centrifuged. The supernatant was collected in another container and evaporated with nitrogen gas in a hood at 40 °C. After this, the dry extracts were reconstituted in 200 µL of H_2_O (pH 3, 0.1% *v/v* CH_3_COOH):CH_3_OH (85:15 *v/v*). During the sample treatment, glass material such as glass tube and Pasteur pipette was used. This solution was analyzed by UPLC-MS/MS. The results were standardized by the protein levels measured in the retinal homogenates using the Pierce BCA protein assay kit (Fisher Scientific, Madrid, Spain) following the manufacturer׳s protocol.

### 2.4. Plasma Sample Analysis

Blood samples were collected through the central atrial artery of the ears in heparinized tubes at the time of sacrifice and were processed within 30 min after extraction. The samples were centrifuged for 10 min at 1000× *g* at room temperature, and the supernatant (plasma) was separated and aliquoted. Thereafter, samples were stored at −80 °C until analysis.

A volume of 200 µL of plasma samples was thawed at 4 °C and 5 µL of internal standard solution (20 µM) was added. Plasma samples were subjected to basic hydrolysis by adding 200 µL of 15% KOH solution (*w/v*). The mixture was incubated at 40 °C for 30 min. Thereafter, samples were diluted with 1360 µL of H_2_O (pH 3, 0.1% *v/v* HCOOH):MeOH (85:15), acidified with 40 µL of formic acid, placed on ice for 10 min, and centrifuged at 4000× *g* for 10 min at 4 °C. The supernatant was collected and a cleaning and preconcentration step was performed using solid phase extraction (SPE). This was performed to achieve a higher concentration of the analytes, using SPE-96 well plates (Discovery^®^ DSC-18, Sigma-Aldrich, St. Louis, MO, USA). SPE cartridges were conditioned first with 1ml of methanol and then with 1 mL of H_2_O (pH 3, 0.1% *v/v* HCOOH). Samples were added in SPE wells, and each well was washed with 500 µL H_2_O (pH 3, 0.1% *v/v* HCOOH) and 500 µL heptane. Elution was carried out with 4 × 100 µL ethyl acetate in 96-well sample plates (Acquity UPLC 700 µL, from Waters, Barcelona, Spain). The recovered samples were evaporated in a speed vacuum concentrator (Savant SPD111V, Thermo Scientific, Waltham, MA, USA) at 45 °C. Finally, the extracts were reconstituted in 60 µL of H_2_O (pH 3, 0.1% *v/v* HCOOH): CH3OH (85:15 *v/v*) and measured in the UPLC–MS/MS.

### 2.5. Urine Sample Analysis

For the analysis of the samples, 1 mL of urine was used. The samples were thawed at 4 °C and centrifuged at 14,000× *g* for 10 min at 4 °C. The supernatant was collected and 5 µL of internal standard solution (20 µM) was added. Then an SPE and elution in ethyl acetate were performed in the same way as for the retina samples in order to achieve a higher concentration of the analytes. Once evaporated, the dry extracts were reconstituted in 100 µL of H_2_O (pH 3, 0.1% *v/v* HCOOH):CH_3_OH (85:15 *v/v*). Finally, they were injected into the chromatographic system (UPLC-MS/MS).

Results were standardized by creatinine levels measured using a creatinine assay (MicroVue creatinine EIA, Quidel Corporation, San Diego, CA, USA) according to the manufacturer’s instructions.

Validation of the bioanalytical method in plasma and urine has been shown in previous studies [16] and the guidelines of the United States Food and Drug Administration were followed [17].

### 2.6. UPLC-MS/MS Analysis

UPLC-MS/MS analysis was carried out on an Acquity-Xevo TQ system (Waters, Barcelona, Spain). The conditions used were: ionization in negative mode (ESI-), capillary voltage 3.5 kV, source temperature 120 °C, desolvation temperature 300 °C, gas flow of the nitrogen cone of 150 L/h, and desolvation flow of 680 L/h.

Separation conditions were selected to achieve appropriate chromatographic retention and resolution by using a C18 column (2.1 × 50 mm, 1.7 μm) (Acquity UPLC BEH) and pre-column (2.1 × 5 mm) from Waters. A binary mobile phase CH_3_OH (0.1% *v/v* HCOOH):H_2_O (0.1% *v/v* HCOOH) with gradient elution was used. The flow rate was 0.4 mL/min, the temperatures of column and the autosampler were 37 °C and 4 °C, respectively. The injection volume was 10 µL. The gradient started with 30% *v/v* CH_3_OH (0.1% *v/v* HCOOH) (i.e., channel B) and from 1 to 4.0 min %B increased up to 90%. Finally, the mobile phase composition returned to the initial conditions at 4.1, and it was maintained for 3.9 min for system conditioning.

The detection was performed by multiple reaction monitoring using the acquisition parameters obtained in a previous study [18,19].

For data acquisition and processing, MassLynx 4.1 and QuanLynx 4.1 softwares from Waters (Waters, Barcelona, Spain) were used, respectively. Linear response curves were calculated employing PGF_2α_-d_4_ as internal standard.

### 2.7. Statistical Analyses

All values in retina, plasma and urine were expressed as median (inter-quartile range, IQR). A one-way ANOVA was employed to determine the differences among groups, followed by Tukey’s multiple comparison test. The null hypothesis was rejected for all the values in the tests in which the F value was significant with a *p*-value less than 0.05. Statistical analyses were performed using Prism 5.0 for Windows software (GraphPad Software, San Diego, CA, USA).

## 3. Results

### 3.1. Pterostilbene Reduces Harmful Retinal Lipid Oxidation Induced by Chronic Hyperglycemia In Vivo

**Figure 2:**
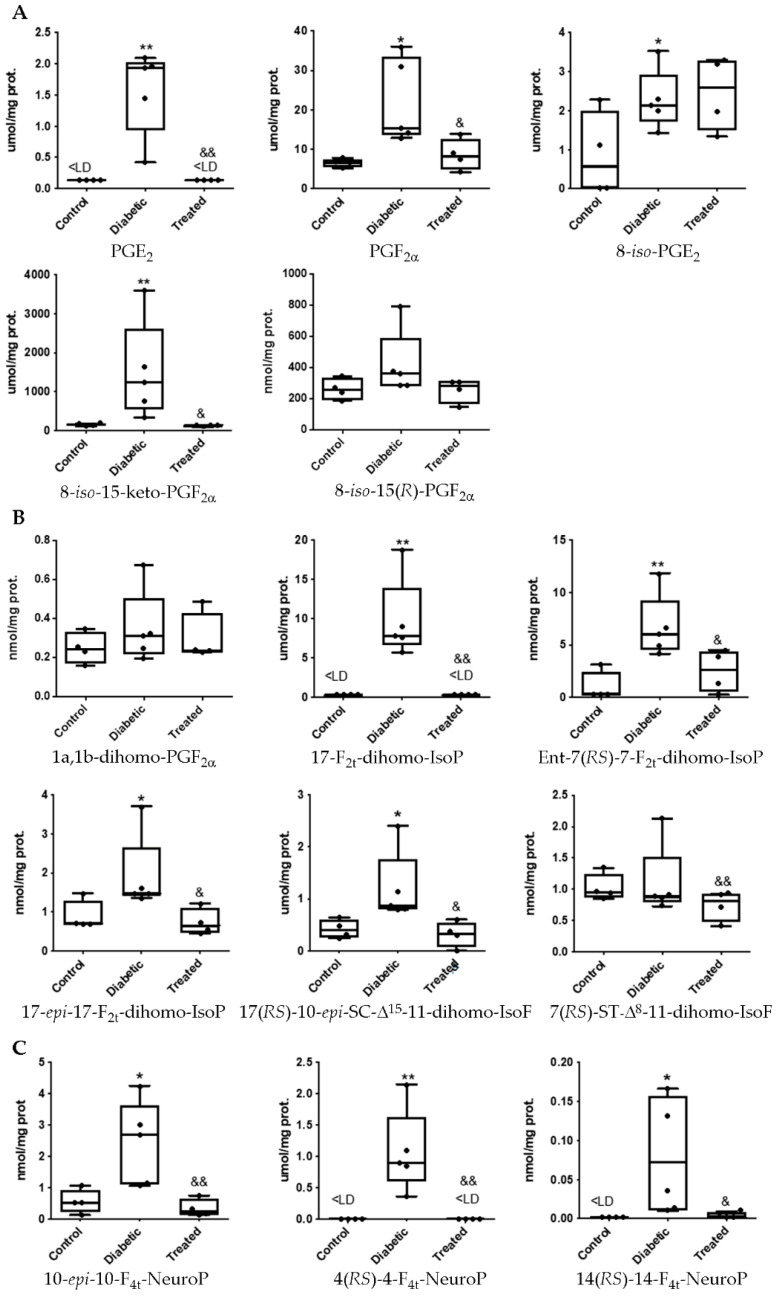
Boxplots of lipoperoxidation analytes in retina samples from control, diabetic and treated group. Lipid peroxidation compounds derived from (**A**) arachidonic acid, (**B**) adrenic acid and (**C**) docosahexaenoic acid. Boxes indicate the 1st and the 3rd quartiles, the median is shown as a black line, whiskers mark the maximum and the minimum values. Values below the limit of detection were replaced by the number of quantification limit indicated by (<LD). The statistical difference is indicated as * < 0.05 vs. control, ** < 0.01 vs. control, & < 0.05 vs. diabetic, && < 0.01 vs. diabetic.

It is well-known that chronic hyperglycemia is a potent inductor of reactive oxygen species. In a recent study, using the current experimental model, we showed that hyperglycemia induces very early lipid oxidation in retina tissue of diabetic animals [7]. In addition, our group showed the ability of Pter to lower lipid peroxidation evaluated by 4-hydroxy-2-nonenal levels in diabetic treated animals [7]. Here we look more in depth at retinal lipids oxidation under diabetes. As shown in Figure 2, chronic hyperglycemia increases lipid peroxidation on arachidonic, adrenic and docosahexaenoic acids in retinal samples. Specifically, we detected significant increases in the values of the prostaglandins PGE_2_ and PGF_2α_, and the IsoPs 8-*iso*-PGE_2_, 8-*iso*-15-keto-PGF_2α_, and 8-*iso*-15-(*R*)-PGF_2α_ (Figure 2A). Oxidation on adrenic acid was reflected by a significant increase in 17-F_2t_-dihomo-IsoP, Ent-7(*RS*)-7-F_2t_-dihomo-IsoP, 17-*epi*-17-F_2t_-dihomo-IsoP, and 17(*RS*)-10-*epi*-*SC*-Δ^15^-11-dihomo-IsoF (Figure 2B). Similar results were obtained for docosahexaenoic acid oxidation which was reflected in an increase of 10-*epi*-10-F_4t_-NeuroP, 4(*RS*)-4-F_4t_-NeuroP and 14(*RS*)-14-F_4t_-NeuroP (Figure 2C). As we expected, subcutaneous daily administration of Pter was able to reverse these early oxidative alterations (Figure 2).

### 3.2. Determination of Lipid Oxidation Analytes in Plasma and Urine in an Experimental Diabetes Type 1 Model

**Figure 3:**
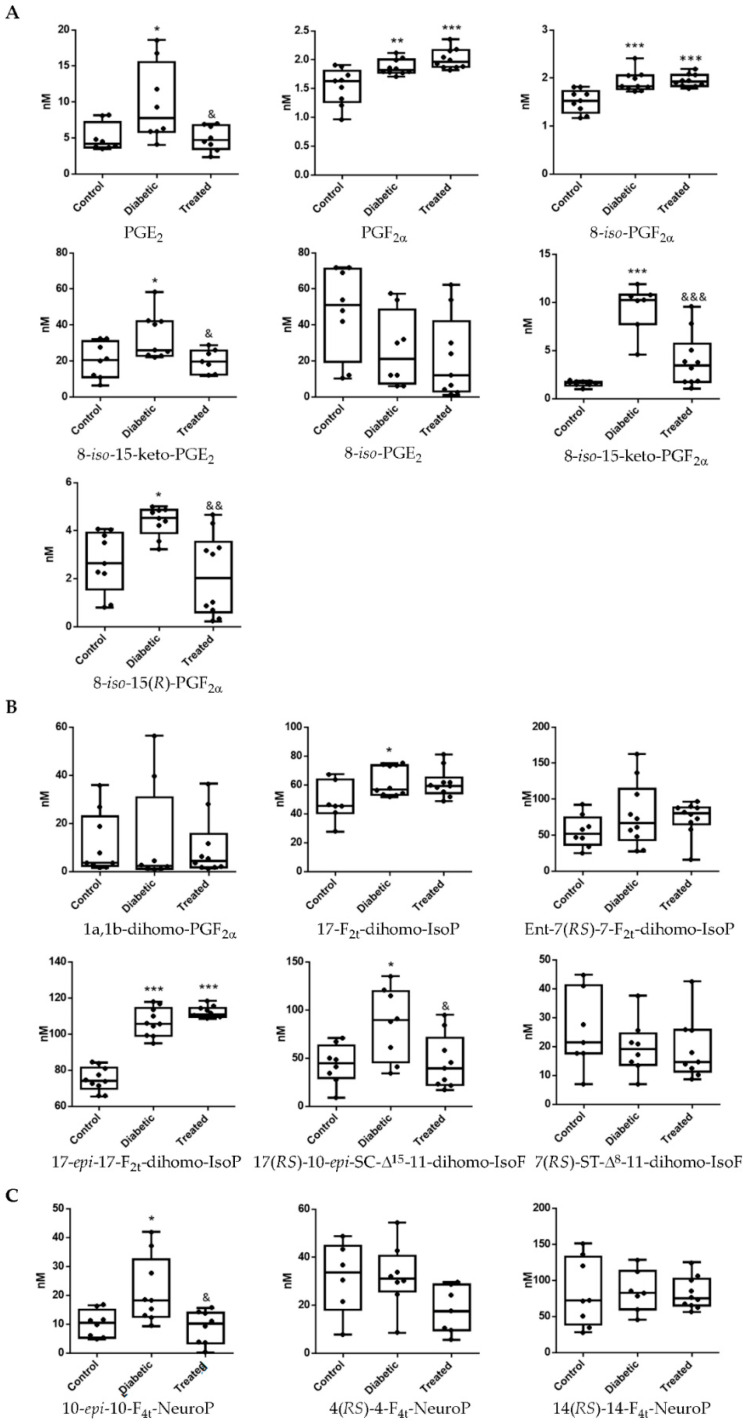
Boxplots of lipoperoxidation analytes in plasma samples from control, diabetic and treated group. Lipid peroxidation compounds derived from (**A**) arachidonic acid, (**B**) adrenic acid and (**C**) docosahexaenoic acid. Boxes indicate the 1st and the 3rd quartiles, the median is shown as a black line, whiskers mark the maximum and the minimum values. Values below the limit of detection were replaced by the number of quantification limit indicated by (<LD). The statistical difference is indicated as * < 0.05 vs. control, ** < 0.01 vs. control, *** < 0.001 vs. control, & < 0.05 vs. diabetic, && < 0.01 vs. diabetic, &&& < 0.001 vs. diabetic.

Diabetes is a systemic disease meaning that it can affect different body organs, and logic suggests that any physiological changes in these organs would be reflected in plasma and urine, since the internal medium is in constant motion between the interstitial fluid and the plasma, as well as in the circulatory system. Hence, analyses of plasma samples clearly showed lipid oxidation levels in diabetic rabbits (Figure 3). Analytes derived from arachidonic acid such as PGE_2_, PGF_2α_, 8-*iso*-PGF_2α_, 8-*iso*-15-keto-PGF_2α_, 8-*iso*-15(*R*)-PGF_2α_ increased (Figure 3A). Likewise, the levels of 8-*iso*-15-keto-PGE_2_, which were non-detected in retina tissue, showed a significant increase in plasma of diabetic animals (Figure 3A). We found that 8-*iso*-PGE_2_ presented undetectable changes between control, diabetic and treated animals (Figure 3A). Lipid oxidation was also reflected in the levels of the adrenic acid derivatives 17-F_2t_-dihomo-IsoP, 17-*epi*-17-F_2t_-dihomo-IsoP, and 17(*RS*)-10-*epi*-*SC*-Δ^15^-11-dihomo-IsoF (Figure 3B) and an increase of docosahexaenoic acid oxidative product 10-*epi*-10-F_4t_-NeuroP in diabetic rabbit plasma samples (Figure 3C).

The antioxidant capability of Pter was demonstrated in a limited number of lipid oxidation analytes in plasma. In Figure 3, we show that treatment with polyphenol is able to lower PGE_2_, 8-*iso*-15-keto-PGE_2_, 8-*iso*-15-keto-PGF_2α_, 8-*iso*-15(*R*)-PGF_2α_ (Figure 3A), 17(*RS*)-10-*epi*-*SC*-Δ^15^-11-dihomo-IsoF (Figure 3B), and 10-*epi*-10-F_4t_-NeuroP (Figure 3C).

**Figure 4:**
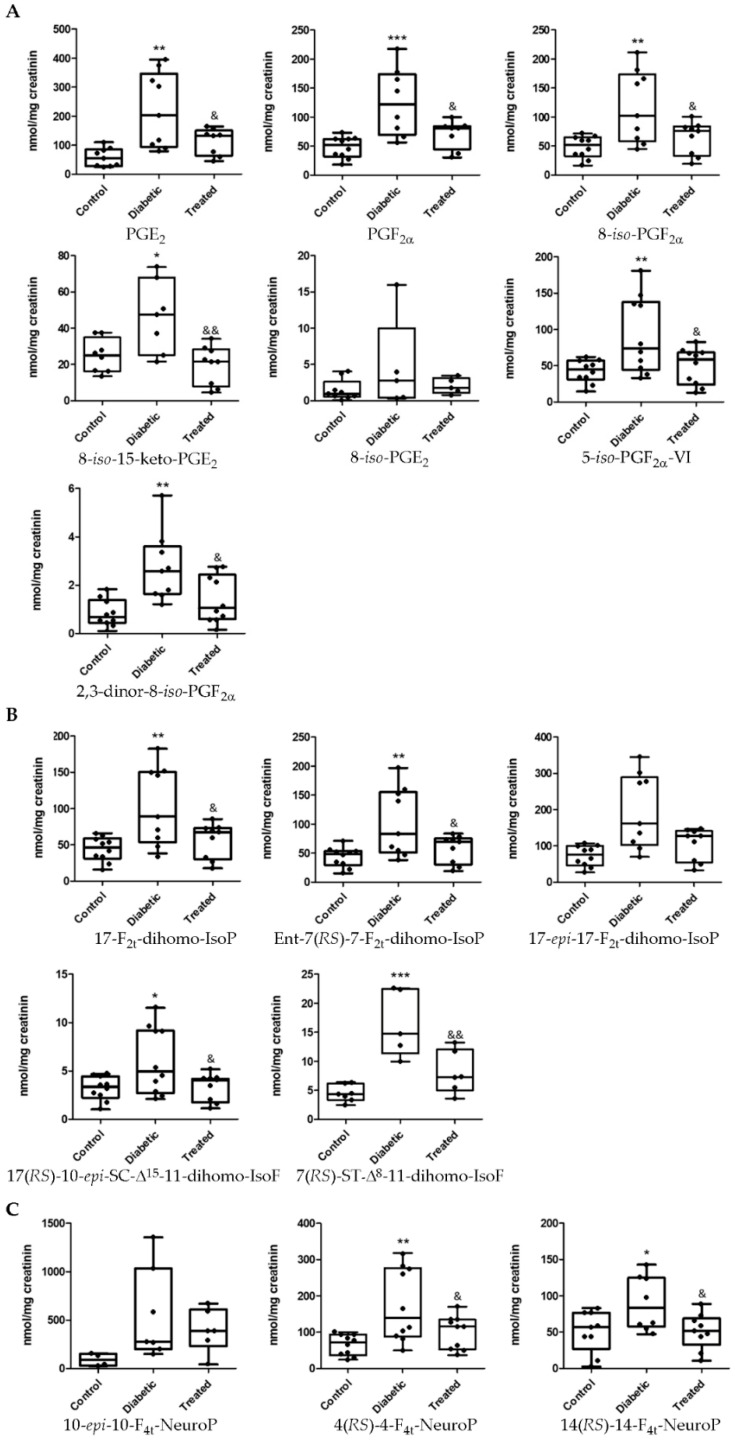
Boxplots of lipoperoxidation analytes in urine samples from control, diabetic and treated group. Lipid peroxidation compounds derived from (**A**) arachidonic acid, (**B**) adrenic acid and (**C**) docosahexaenoic acid. Boxes indicate the 1st and the 3rd quartiles, the median is shown as a black line, whiskers mark the maximum and the minimum values. Values below the limit of detection were replaced by the number of quantification limit indicated by (<LD). The statistical difference is indicated as * < 0.05 vs. control, ** < 0.01 vs. control, *** < 0.001 vs. control, & < 0.05 vs. diabetic, && < 0.01 vs. diabetic.

Diabetes-induced lipid oxidation was reflected in urine in an increase of derivatives of arachidonic acid: PGE_2_, PGF_2α_, 8-*iso*-15-keto-PGE_2_, 8-*iso*-PGF_2α_, 5-*iso*-PGF_2α_-VI, and 2,3-dinor-8-*iso*-PGF_2α_ were increased (Figure 4A). In addition, the levels of the adrenic acid products 17-F_2t_-dihomo-IsoP, Ent-7(*RS*)-7-F_2t_-dihomo-IsoP, 17(*RS*)-10-*epi*-*SC*-Δ^15^-11-dihomo-IsoF, and 7(*RS*)-ST-Δ^8^-11-dihomo-IsoF were also increased (Figure 4B). In a similar way, lipid peroxidation of docosahexaenoic acid induced an increase of 14(*RS*)-14-F_4t_-NeuroP and 4(*RS*)-4-F_4t_-NeuroP levels in urine samples (Figure 4C). The levels of all analytes with significant diabetes-induced changes were normalized with the polyphenol treatment (Figure 4).

## 4. Discussion

DR is considered a multifactorial disease [3]. In fact, it is difficult to understand the exact mechanisms by which diabetes induces DR due to its complex etiology. However, it is well known that chronic exposure to hyperglycemia induces an increase in the production of reactive oxygen species with the subsequent loss of redox homeostasis, which notably contributes to early neuronal retinal cell death [3], pericytes demise, later rupture of the blood retinal barrier, increased vascular permeability [20] and the progression to advanced DR stages [20,21,22]. Delayed treatment of diabetes and cumulative molecular damage from oxidative stress led to the development of DR [23]. It has been shown that, after diagnosis of diabetes, strict and early control of blood glucose and/or dyslipidemia slows the evolution of this microangiopathy [24]. In spite of this, the treatment for DR is restricted to advanced stages of the disease when there are serious vascular alterations and the retina shows neuronal irreparable damages [3]. Therefore, it is imperative to find new strategies for early use to diagnose and prevent DR progression.

For the maintenance and development of the functions of the retina there are a series of essential molecular components such as molecules of long chain polyunsaturated fatty acids (PUFAs), which form part of the cell membranes. In fact, retinal membrane phospholipids contain the highest level of PUFA of any tissue. Specifically, docosahexaenoic acid (DHA; 22:6n-3), arachidonic acid (AA; 20:4n-6) and adrenic acid (22:4n-6) are the most abundant fatty acid in the retina [25,26,27]. Their physiological function include critical protective effects against different retinal diseases such as retinopathy of prematurity, age-related macular degeneration or DR [28]. For example, NPD1, a docosatriene derivative from docosahexaenoic acid, protects retinal epithelial cells from oxidative stress thanks to its anti-apoptotic effects [29], or their metabolism alteration by peroxisome dysfunction induces retinal defects [30].

Polyphenols are the most abundant antioxidants in the human diet [31] and their beneficial effects have prompted interest in their use for treating retinal pathologies. For example, a protective effect has been observed against DR and other retinopathies with curcumin [32,33], resveratrol [34,35,36], quercetin [37,38], epigallocatechin gallate [39,40], and Pter [7], among others. The all have one thing in common, their antioxidant capacity against oxidative stress after hypoxia [41,42] and their significant ability to stop the pathological angiogenesis [43] by reducing levels of the vascular endothelial growth factor (VEGF) [44]. It has therefore been suggested that polyphenolic administration can protect against the development of retinal diseases such as, DR.

Recently, we showed for the first time that daily Pter administration at non-toxic and biologically effective doses of 50 mg/kg can prevent the early neuro-retinal damage caused by hyperglycemia in vivo [7]. It is known that retinal neural cell death appears in early stages of the development of DR, even prior to the appearance of the classic phenotypic characteristics used in its diagnosis [7,45,46,47]. In addition, the increase of lipid peroxidation products has been associated with neurodegeneration diseases [48] and there is increasing evidence of the importance of products of lipid peroxidation as mediators in the development of neovascularization in DR [49,50]. In our previous study, we showed that Pter reduces the levels of 4-HNE [7], an end products of lipid peroxidation of PUFAs such as linoleic acid and arachidonic acid via non-enzymatic steps [27,51]_._ The low levels of 4-HNE induced by the polyphenol can reduce the former’s damaging effects on proteins, RNA and DNA synthesis [52]. Moreover, Aguirre et al. showed that Pter mainly increases docosahexaenoic acid levels, although linoleic acid and arachidonic acid also increase without reaching statistical significance, indicating the ability of stilbene to restore the PUFA composition in a model of hepatic steatosis [53]. This ability of Pter could be relevant in the antioxidant protection observed in our experimental model.

The role of lipid peroxidation in DR has been extensively studied [54,55,56] but in a general way, focusing on the manifestation of a limited number of products such as MDA, 8-Iso-PGF_2α_ or 4-HNE, without focusing on the damage of omega-3 PUFA in such a specific way. Little is known about the effect of the protective role of polyphenols against radical induced peroxidation of omega-3 PUFAs by ROS related to neurodegeneration and its implication in the development of DR. In this research, an exhaustive study of the damage produced by oxidative stress in lipids of the retina was carried out. The components studied can be classified depending on the modified lipid from which they are derived. We studied a group of products derived from the oxidation of arachidonic acid, docosahexaenoic acid, and adrenic acid (Figure 1). Spectrometry analyses showed that lipid peroxidation in the retinas of diabetic rabbits increased the levels of: PGE_2_, PGF_2α_, IsoPs 8-*iso*-PGE_2_, 8-*iso*-15-keto-PGF_2α_, and 8-*iso*-15-(*R*)-PGF_2α_, 17-F_2t_-dihomo-IsoP, Ent-7(*RS*)-7-F_2t_-dihomo-IsoP, 17-*epi*-17-F_2t_-dihomo-IsoP, 17(*RS*)-10-*epi*-*SC*-Δ^15^-11-dihomo-IsoF, 10-*epi*-10-F_4t_-NeuroP, 4(*RS*)-4-F_4t_-NeuroP, and 14(*RS*)-14-F_4t_-NeuroP. The action of the natural polyphenol Pter was able to restore to control values the levels in diabetic rabbits (Figure 2).

The biological effect on DR of some of these compounds is well establish and the protector role of this stilbene against DR progression has attracted much attention. The formation of prostaglandins, inflammatory mediators synthetized from arachidonic acid by cyclooxygenase enzyme (COX), such as PGE_2_ and PGF_2α_ are associated with retinal vasoconstriction, blood ocular barrier disruption, VEGF production, increased vasodilation, leukocyte migration, and increased production of proinflammatory cytokines [49,57,58,59,60,61]. For example, the extracellular signal-regulated kinases 1 and 2 (ERK1/2)/COX-2/PGE_2_ signaling pathway induces the expression of VEGF [49], whose overexpression is related to the vascular hyperpermeability and neovascularization developed in retinas of diabetic patients [3]. Moreover, progression of DR may be prevented or delayed by the use of prostaglandin inhibitors [49,50,62,63] and COX inhibitors reduce the production of VEGF, vascular leakage and neovascularization in DR and ischemic proliferative retinopathy in vivo [50,64].

Although the effects on DR have not been clearly identified in the present experimental model, from the set of compounds analyzed with significant differences and affected by Pter treatment we can make some interesting observations. 8-*iso*-15-Keto-PGF_2α_ is a metabolite of the 8-*iso*-PGF_2α_, a prostaglandin-like product produced by the non-enzymatic peroxidation of arachidonic acid in membrane phospholipids [65] that showed vasoconstrictor effects in a concentration-dependent manner in rat aorta [66] and has been proposed as a biomarker for ischemic stroke diagnosis [67]. 8-iso-PGE_2_ is also an isoprostane produced from arachidonic acid during lipid peroxidation [68] with renal vasoconstrictor effects in rat [68,69] and its urinary quantification is considered a reliable marker of systemic oxidative stress, even superior to its plasmatic evaluation [54]. Ent-7(*RS*)-7-F_2t_-dihomo-IsoP, 17-*epi*-17-F_2t_-dihomo-IsoP, 17-F_2t_-dihomo-IsoP formed by a free radical non-enzymatic mechanism from adrenic acid (C22:4 n-6, AdA) [70] and, 10-*epi*-10-F_4t_-NeuroP, 4(*RS*)-4-F_4t_-NeuroP, and 14(*RS*)-14-F_4t_-NeuroP by docosahexaenoic acid (C22:6 n-3, DHA) [71], have been used as biomarkers of oxidative stress in neurodegenerative disease [72,73,74,75,76]. Hence, although further research is necessary to clarify the pathophysiological action triggered by those PUFA derivatives in DR, Pter is able to normalize the levels of almost all analytes studied in retinas of diabetic rabbits, helping to protect the retina and avoiding the appearance of the very early signs of DR such as neuronal cell demise derived from oxidative stress.

Furthermore, the results presented allow us to hypothesize about the possible use of lipid peroxidation as an early biomarker of diabetic retinal disease. The diagnosis of DR and its ophthalmologic classification is based on the results of the multicenter Early Treatment Diabetic Retinopathy Study (ETDRS). This study classified DR in function of the visible ophthalmologic alterations and retinal neovascularization development [3,77,78]. There is no question of the usefulness of this diagnostic method in delaying disease progression to the sight loss. However, although the ophthalmologic diagnosis is minimally invasive, rapid and economical, the phenotypic manifestation of the disease involves the development of previous molecular unobservable alterations that trigger neuronal and vascular damage. Hence, because DR is an asymptomatic disease until reaching advanced stages, the identification of early diagnosis biomarkers could be the best tool to prevent the progression of the disease to an irreversible stage and ultimately the loss of vision. Therefore, analysis of these peroxidation lipid analytes at plasma and urine levels could instantly provide a clinical reflection of the oxidative status of patients before and after specific clinical interventions. In Figure 3 and Figure 4, a comparative study on lipid peroxidation analytes in plasma and urine samples between control, diabetic and Pter diabetic treated rabbits are shown. The oxidative environment induced by hyperglycemia together with the presence of skipped dienes makes PUFAs highly susceptible to oxidation [79,80]. PUFA-containing phospholipids react with reactive oxygen species generating a variety of oxidized products potentially harmful and capable of acting on targets located at a certain distance from the initial oxidative attack, since some of these products are more durable than reactive oxygen species [27,81]. Therefore, the measurement of concentrations of analytes of lipid peroxidation in biological samples is considered an important tool for evaluating the role of oxidative processes in the pathogenesis of human diseases and the response to specific therapies [16]. In addition, the study of these analytes in fluids such as plasma and urine can instantly provide a clinical reflection of the oxidative state and are useful in patients studies before and after specific clinical interventions (i.e., antioxidant treatment).

A number of published works indicate that oxidative stress caused by diabetes triggers lipid alterations with serious pathophysiological effects that contribute to the development of DR both in animal models [82] and in the retina of diabetic patients [83]. Moreover, patients suffering DR show higher lipid peroxidation than those without the retinal disease [84,85]. Although diabetes is associated with increased systemic oxidative stress, the present study demonstrates the ability of Pter to lower lipid peroxidation detected in plasma and urine and suggests new potential biomarkers to predict DR progression. Moreover, the results presented herein show a deeper and more extended lipid peroxidation study beyond the classic MDA or 4-HNE.

One of the most in vivo produced and studied F_2_-IsoP is 8-iso-PGF_2α_. In fact, it has been considered the gold standard biomarker of in vivo oxidative stress. However, we did not detect significant alterations in retina tissue (Figure 2). Different results were obtained from plasma and urine of diabetic rabbits, where levels of this F_2_-IsoP increased (Figure 3 and Figure 4). Although 8-iso-PGF_2α_ is formed from arachidonic acid predominantly via non enzymatic oxidation, it can be generated by COX activity through the enzymatic oxidation of PGF_2α_ [86]. COX activity is ubiquitously induced in diabetic patients and F_2_-IsoPs have been involved in different acute and chronic human diseases related to oxidative and inflammatory stress such as diabetes [87]. This situation rules out considering 8-iso-PGF_2α_ as a suitable biomarker for DR.

One approach that could help support our hypothesis is to investigate the location of production of the different lipid peroxidation products studied. The products studied derived from arachidonic acid oxidation show a ubiquitous distribution in the body tissues, including retina [55]. The oxidation of docosahexaenoic acid and the production of NeuroP/neurofurans occurs mainly in brain grey matter and retina [71,88,89], and dihomo-IsoP/dihomo-IsoF from adrenic acid oxidation are found in brain white matter and retina [15,70,90,91]. Furthermore, although it has not been studied in depth, docosahexaenoic and adrenic acid have been considered specific tissue markers of oxidative damage in neurological disorders such as Rett syndrome, Down syndrome, epilepsy, Alzheimer’s disease and age-related macular degeneration [48,92]. Hence, we consider that lipid peroxidation compounds derived from docosahexaenoic and adrenic acid may be the most interesting analytes in the study of development and progression of DR. We detected parallel lipid peroxidation in retina and urine samples induced by hyperglycemia and the reinstatement to control values with the polyphenol treatment in 17-F_2t_-dihomo-IsoP, Ent-7(*RS*)-7-F_2t_-dihomo-IsoP, 17(*RS*)-10-*epi*-*SC*-Δ^15^-11-dihomo-IsoF, 4(*RS*)-4-F_4t_-NeuroP (Figure 2 and Figure 4). In plasma and retina samples, we detected similar alterations just in 17(*RS*)-10-*epi*-*SC*-Δ^15^-11-dihomo-IsoF and 10-*epi*-10-F_4t_-NeuroP (Figure 2 and Figure 3). Focusing on 17(*RS*)-10-*epi*-*SC*-Δ^15^-11-dihomo-IsoF, which is derived from adrenic acid, this is increased in the retina, plasma and urine of diabetic rabbits and Pter is able to lower its concentration. Although further research with human samples in different DR evolution stages will help to corroborate its importance and its role, for the first time 17(*RS*)-10-*epi*-*SC*-Δ^15^-11-dihomo-IsoF is shown as a possible suitable biomarker of DR in the prevention of the development of the pathology.

## 5. Conclusions

Our study demonstrates the significant ability of Pter to prevent the retinal early lipid peroxidation induced in vivo by hyperglycemia, phenomenon determinant in the development and evolution of DR. A large group of specific neuronal and retinal lipid peroxidation markers was studied including derivatives from adrenic and docosahexaenoic acid oxidation in plasma and urine samples. 17(*RS*)-10-*epi*-*SC*-Δ^15^-11-dihomo-IsoF, a product derivative from adrenic acid oxidation, is postulated for the first time as an early DR biomarker. Further studies will be carried out with human samples at different stages of evolution to validate the usefulness of the determination of lipid peroxidation in plasma and/or urine in the diagnosis and staging of DR.

## Data Availability

The data presented in this study are available within the article.
